# Therapeutical and Pharmaceutical Notes on Cimicifuga

**Published:** 1861-03

**Authors:** Edward Parrish


### *Therapeutical and Pharmaceutical Notes on Cimicifuga.* By Edward Parrish.

At the risk of being charged with traveling out of the legitimate sphere of the pharmaceutist, I offer to the profession the following notes on one of the most valuable of our indigenous drugs.

The recent admirable remarks on cimicifuga, by Prof. Simpson, of Edinburgh, published in the London *Medical Times & Gazette*, have reminded me of several unpublished cases, in which it has been used with great success. One of these occurred in the practice of my friend and pupil in pharmacy, Dr. Charles Schaffer, who employed the resinoid active principle, known in commerce as cimicifugin, at my suggestion, in an anomalous case, which, with the other treatment, I shall describe as related to me.

The patient was a lady of about forty-six years of age, thin and anæmic, of highly nervous temperament, yet so weak as to be unable to walk a quarter of a mile without suffering great fatigue; her appetite was exceedingly poor, almost amounting to loathing of food; she often ate nothing at breakfast and but little at other meals; her urine was observed to be frequently loaded with uric acid and urate of ammonia, especially after undue exercise. She had been partially in this condition for a number of years, but within the few months preceeding, suffered from the additional trouble of wakefulness, which now amounted to a serious matter, as she would sleep but three or four hours out of the twenty-four, and, of course, suffered the consequences; this last symptom had, probably, some connection with mental disturbance, caused by the recent death of an intimate friend.

For these symptoms, and especially the great lassitude and weakness, she was freely treated with tonics and stimulants, such as proto-carbonate of iron and quassia, and, at bed-time, Dover's powder, etc., with very partial success, the morbid vigilance seeming to be aggravated by the stimulating action of the opiates, although sleep was frequently obtained the next day as a result of the great weariness.—Valerian, asafœtida, chloroform, ether, and conium, were also resorted to, with neither complete nor permanent success; chloroform gave the best results of any of the sedatives, but it produced such nausea that the patient was obliged to discontinue it. Sponging with whisky and the use of syrup of wild cherry, with small doses of hydrocyanic acid, at retiring, were the most effectual palliatives for the wakefulness, and the use of solution of caustic potassa diminished the urinary deposits; but there was felt to be a want of some remedial agency to reach the *cause* of the unusually persistent symptoms. The resin of cimicifuga was employed to this end, beginning with quarter-grain doses, three or four times a day, according to circumstances; this, though occasionally producing headache, was immediately successful, inducing quiet sleep, restoring the appetite, and gradually diminishing the urinary deposit.

Iron which had been used at intervals from the first, was now combined with the resin; the health of the patient continued to improve; at the date of this information she could walk a mile or more without unpleasant fatigue; loses but one or two hours of the nine or ten allotted to sleep, and that from the habitual dread of wakefulness rather than from any physical cause.

The results, in this case, seem to indicate a trial of cimicifuga in anomalous cases of nervous disorder resisting ordinary stimulant and sedative treatment, and the many cases of chorea and rheumatism on record in which it has been found effectual, the testimony now coming across the water from the distinguished Edinburgh Professor, who has used it successfully in puerperal hypochondriasis, must draw increased attention to it as filling up a gap in the materia medica. Of the drug itself too little is known by practitioners, who, in their daily rounds, pass by its nodding racemes projecting above the fence tops, from Canada to Florida, without the least chagrin that some of its most valuable adaptations should be first brought to notice in a foreign land. This root is well described by Dr. Wood in Dispensatory, but one remarkable character has not been mentioned in any of the descriptions which I have seen ; it is the peculiar appearance of the cross section of the rootlets; owing to the irregular shrinkage of the central ligneous portion, which is of a light color, while the external layers are nearly black, the fracture displays a star-shaped or lobed figure, frequently with five divisions, though sometimes with three, and often four resembling the Maltese cross. The rough and contorted head forms by far the largest part of the drug as found in the shops; it is hard and resinous, and possesses a peculiar though not very strong odor, which is lost by age and deterioration. This drug is so little called for in the shops that it is generally laid away on a shelf till it is perhaps covered with mould, and has lost any volatile ingredient which may be supposed to add to its efficiency. Let this fact have its weight in estimating its efficiency from past experience.

The preparations in use are the powder, very ineligible, requiring to be given in 20 to 60 grain doses. The tincture which, though not officinal, is made usually in about the proportion of two ounces to the pint of diluted alcohol, and given in a dose of a teaspoonful or more. The fluid extract which, like most of this class of preperations, is designed to represent the drug in the proportion of a fluid ounce to each ounce, and is appropriately given in the dose of 30 drops. The solid extract, which is also unofficinal, and seldom met with, is well adapted to the pillular form, eight grains representing a drachm of the root; dose 3 to 8 grains.

The so-called active resinoid principle cimicifugin or macrotin, as called by some manufacturers, originated with the Western School of Eclectics, who carry a certain method of preparation to a ridiculous extreme, using it for nearly all drugs. This method consists in thoroughly exhausting the drug by strong alcohol, concentrating to a syrupy consistence, and precipitating by water; a process which seems well adapted to this and several other drugs. As thus prepared, cimicifugin is a dry, insoluble powder, active in doses of a quarter to half a grain, sometimes increased to one grain, and, excepting in the partial loss of the volatile ordorous principle, it seems a good representative of the root itself.—*Philadelphia Reporter.*

